# Comparative Analysis of Coagulation and Liver Parameters in Individuals with Alcohol and Substance Use Disorders and Healthy Controls

**DOI:** 10.3390/diagnostics16010052

**Published:** 2025-12-23

**Authors:** Şeyma Bardakçı, Muhammed Raşit Bardakçı, Derya Güzel Erdoğan, Abdülkadir Aydın, Ahmet Bulent Yazici

**Affiliations:** 1 Department of Physiology, Faculty of Medicine, Sakarya University, 54100 Sakarya, Turkey; seymaisikanbardakci@gmail.com (Ş.B.); deryaguzel@sakarya.edu.tr (D.G.E.); 2 Clinic of Psychiatry, Sakarya Training and Research Hospital, 54100 Sakarya, Turkey; drmrb0209@gmail.com; 3 Department of Family Medicine, Faculty of Medicine, Sakarya University, 54100 Sakarya, Turkey; abdulkadiraydin@sakarya.edu.tr; 4 Department of Psychiatry, Faculty of Medicine, Sakarya University, 54100 Sakarya, Turkey

**Keywords:** alcohol, coagulation, liver functions, substance

## Abstract

**Background/Objectives:** Alcohol Use Disorder (AUD) and Substance Use Disorders (SUDs) can affect both the liver, where clotting factors are synthesized, and the coagulation system, which prevents acute bleeding. **Methods:** This study included 451 inpatients undergoing addiction detoxification and 150 healthy controls. Patients were stratified by substance type: Alcohol (*n* = 110), Cannabinoid (*n* = 71), Methamphetamine (*n* = 110), Multiple-Substance (Methamphetamine + Cannabinoid, *n* = 110), and Opioid (*n* = 50) users. Age-matched control groups (mean ages 45, *n* = 50; 30, *n* = 100) were used. Serum levels of Ca, INR, PT, APTT, PLT, AST, and ALT, alongside sociodemographic variables, were assessed. **Results:** Significant group differences were observed in ALT, AST, PT, APTT, and PLT (*p* < 0.001). Notably, PT was lower in Multiple Substance and Methamphetamine users; APTT was elevated in Cannabinoid users; AST was higher in Alcohol users; and Methamphetamine and Opioid users exhibited both decreased AST and ALT. Post hoc analyses confirmed substance-specific effects (*p* < 0.001). Regular cigarette use was significantly more prevalent among alcohol and substance user groups compared to controls; however, smoking did not exert a significant effect on the evaluated biochemical or coagulation parameters. **Conclusions:** These findings demonstrate that liver enzymes and coagulation parameters can vary significantly by substance type. Observed alterations in AST, ALT, PT, APTT, and PLT suggest that substance use may exert substance-specific effects on hepatic and haemostatic function, highlighting potential risks for bleeding or thrombotic complications. Monitoring these parameters in AUD and SUD patients could provide valuable clinical insights, allowing for more tailored and proactive management strategies. While the underlying mechanisms remain to be fully elucidated, these results emphasize the importance of considering substance-specific physiological impacts when assessing liver and coagulation health in addicted populations.

## 1. Introduction

Coagulation is a critical physiological process essential for maintaining vascular integrity and preventing acute hemorrhage through the intricate interactions among platelets and various clotting factors [1]. The haemostatic process is initiated following vascular injury, which prompts rapid platelet adhesion, activation, and aggregation, subsequently leading to the accumulation of clotting factors that serve to stabilize the platelet plug. Disruptions to this finely tuned process can result in an increased risk of both bleeding and thrombotic events [2].

The liver plays a pivotal role in the synthesis of prothrombin, fibrinogen, and other coagulation proteins that are essential for effective haemostasis. Dysfunctional liver activity can lead to a diminished production of these proteins, thereby prolonging prothrombin time (PT) and activated partial thromboplastin time (aPTT) [3,4]. Both PT and aPTT are widely used to assess coagulation pathways and haemostatic function, with PT often converted to the international normalized ratio (INR) to standardize results and monitor vitamin K antagonist therapy such as warfarin [5,6].

Alcohol Use Disorder (AUD) and Substance Use Disorders (SUDs) are increasingly prevalent psychiatric conditions influenced by a myriad of psychosocial and biochemical factors [7,8,9]. It is known that alcohol and substance use affect the coagulation system through various mechanisms. Studies on the effects of alcohol on coagulation factors have associated chronic alcohol use with impaired platelet aggregation and compromised liver function, which may lead to decreased production of coagulation factors and increased risk of bleeding [10,11]. In contrast, a cohort study by Shen et al. showed that alcohol consumption, especially when liver dysfunction develops, can also affect the synthesis of anticoagulant factors, leading to a prothrombotic state [12]. Studies have shown that amphetamines may cause disruption in blood coagulation mechanisms through effects such as oxidative stress, endothelium damage associated with MAPK activation, and increased plasma α2-macroglobulin and fibrinolysis [13,14]. Substances such as morphine and nicotine, which are subject to misuse, have been reported to activate postsynaptic dopamine receptors, leading to an increase in extracellular tissue-type plasminogen activator (tPA) activity and thereby affecting the coagulation system [15,16]. A review study by Carter et al. indicates that cannabis use has effects on platelet activation and aggregation [17]. Previous studies indicate that alcohol and other psychoactive substances exert distinct impacts on liver function, underscoring the necessity of evaluating substance-specific differences within clinical populations [18,19]. Furthermore, coagulation and liver function may be influenced not only by substance use but also by underlying infectious conditions such as HIV, HBV, and HCV infections, as well as metabolic factors like calcium imbalances, all of which are critical clinical considerations when interpreting laboratory results [20].

As seen in the literature, studies on this subject have not evaluated the effects of alcohol or drugs on liver enzymes or coagulation parameters within a combined clinical framework; generally, each substance has been examined separately. In our study, we aim to contribute to the existing literature by comparing five different substance use groups with each other and with two age-matched healthy control groups. The evaluation of multiple substance-use groups within a unified cross-sectional study facilitates direct comparisons and enhances the understanding of substance-specific effects on liver function and coagulation parameters. This approach addresses a notable gap in the literature, where most existing studies tend to evaluate these factors in isolation.

Considering the rising prevalence of AUD and SUD and their potential repercussions on haemostasis and liver function, this study seeks to assess coagulation parameters (PT, aPTT, platelet count) and liver enzymes (AST, ALT) in individuals with AUD or SUD based on the specific substance used, taking into account possible confounding factors (sociodemographic characteristics, smoking, HIV-HBV-HCV infection, calcium level, etc.), compared with healthy control groups. The findings aim to elucidate substance-specific variations in coagulation and liver function, thereby contributing valuable insights that may inform clinical monitoring and risk assessment strategies.

## 2. Materials and Methods

### 2.1. Study Design and Sample

In this retrospective study, the medical records of patients admitted to the Sakarya University Training and Research Hospital Inpatient Detoxification Center between 1 January 2022 and 31 January 2025 were analyzed. In this unit, admission is based on routine clinical criteria that require the absence of acute intoxication, defined as a breath alcohol level of ≤0.5% for alcohol users and the lack of clinically observable psychoactive effects for individuals using other substances. In this way, the laboratory measurements obtained on the first day of hospitalization predominantly reflect chronic or subacute changes associated with substance use rather than the transient effects of acute intoxication on liver and coagulation parameters. The control group consisted of healthy volunteers who presented to family medicine outpatient clinics for routine periodic or medical report examinations. Ethical approval was obtained from the Ethics Committee for Scientific Research in Health Sciences at Sakarya University Faculty of Medicine (20 February 2025, E-43012747-050.04-451173-124).

In total, data from 601 participants were included in the study, comprising five diagnostic groups meeting DSM-5 criteria for Substance Use Disorders (SUDs)—Alcohol Use Disorder (*n* = 110), Cannabinoid Use Disorder (*n* = 71), Methamphetamine Use Disorder (*n* = 110), Multiple-Substance Use Disorder (methamphetamine + Cannabinoid; *n* = 110), and Opiate Use Disorder (*n* = 50)—and two age-matched healthy control groups (mean age ≈30 years, *n* = 100; mean age ≈45 years, *n* = 50). Group sizes were based on the distribution of available clinical data and selected to maintain comparable numbers across diagnostic categories, ensuring adequate representation for statistical comparisons. A post hoc sensitivity analysis using G*Power 3.1 was performed to assess sample adequacy. Assuming a one-way ANOVA with seven groups, an α = 0.05, and total *n* = 601, the analysis indicated a statistical power (1 − β) of approximately 0.92 for a moderate effect size (f = 0.25) and greater than 0.80 for smaller effects (f ≈ 0.18), based on conventional benchmarks proposed by Cohen [21]. These findings confirm that the study sample was sufficient to detect meaningful between-group differences. All individuals diagnosed with SUD, including AUD, were regular smokers, while the majority of control participants were also regular smokers. Inclusion criteria for the study required participants to be between 18 and 65 years of age, without a history of schizophrenia, other psychotic disorders, bipolar disorder, intellectual disability, severe neurological or metabolic disease, anticoagulant medication use within the past year, or analgesic/anti-inflammatory medication use within the past month. Healthy control participants were additionally required to have no history of Alcohol or Substance use disorders and no regular use of alcohol or illicit substances.

### 2.2. Coagulation Parameters and Liver Function Tests

Sociodemographic characteristics (age, gender) and laboratory data—including serum calcium, PT, aPTT, INR, PLT, AST, and ALT—were obtained from patient records and routine laboratory tests performed at admission to the inpatient detoxification unit, ensuring confidentiality. All biochemical analyses for both patient and control groups were performed in the same hospital laboratory using standardized and quality-controlled automated assays, with patient samples collected on the first day of admission to the detoxification unit, prior to the initiation of pharmacological or supportive treatment interventions, thereby reflecting baseline physiological status. Participants were also evaluated for HBV, HCV, and HIV seropositivity. Laboratory and sociodemographic parameters were compared both across substance-use groups and between patient and control groups to evaluate differences in coagulation and liver function. Potential confounders such as age and sex were statistically controlled during group comparisons to ensure standardized and reliable results.

### 2.3. Statistical Analysis

The data collected for this study were analyzed using IBM SPSS Statistics version 26.0. Descriptive statistics, including means, standard deviations, and frequencies, were calculated for all variables. To assess the normal distribution of continuous variables, skewness and kurtosis statistics were evaluated, with values ranging from −2 to +2 indicating that the assumptions of normality were satisfied [22]. Two-group comparisons were performed using the Mann–Whitney U test for non-normally distributed data, whereas one-way ANOVA with Bonferroni post hoc tests or Kruskal–Wallis tests with pairwise comparisons were used for multi-group analyses depending on data normality. Categorical variables were analyzed using the Chi-square test or Fisher’s exact test, as appropriate. When significant associations were detected, post hoc analyses based on adjusted residuals and Bonferroni-corrected pairwise comparisons were conducted to identify the source of group differences. Results are presented with 95% confidence intervals, and a *p*-value < 0.05 was considered statistically significant.

## 3. Results

### 3.1. Demographic Characteristics Between Substance Abusers and Control Groups

When the groups were compared in terms of average age, a significant difference was found between the groups (F = 79.456, *p* = 0.000) (Table 1). In the post hoc analysis, the difference in age averages was found to be significant between the 45-year-old control and alcohol group and the other groups (*p* < 0.001). The 30-year-old control group showed no significant differences between methamphetamine, cannabinoid, opioid, and multiple substance groups. (*p* > 0.05).

When groups were evaluated by gender, no significant differences were found between groups (*p* > 0.05).

Data are presented as mean ± standard deviation for continuous variables and as numbers (percentages) for categorical variables. Group differences were assessed using one-way ANOVA for continuous variables and the Chi-square test for categorical variables. Statistically significant results are shown in bold (*p* < 0.05).

### 3.2. Comparison of Liver Functions and HBV, HCV, HIV Between Substance Abusers and Control Groups

When the groups were compared in terms of ALT levels, a significant difference was observed (H = 47.354, *p* < 0.001). Post hoc pairwise analyses revealed statistically significant differences between the opioid and 30-year-old control, opioid and alcohol, methamphetamine and 30-year-old control, methamphetamine and alcohol (all *p* < 0.001), and multiple-substance and alcohol (*p* = 0.001) groups.

**Figure 1:**
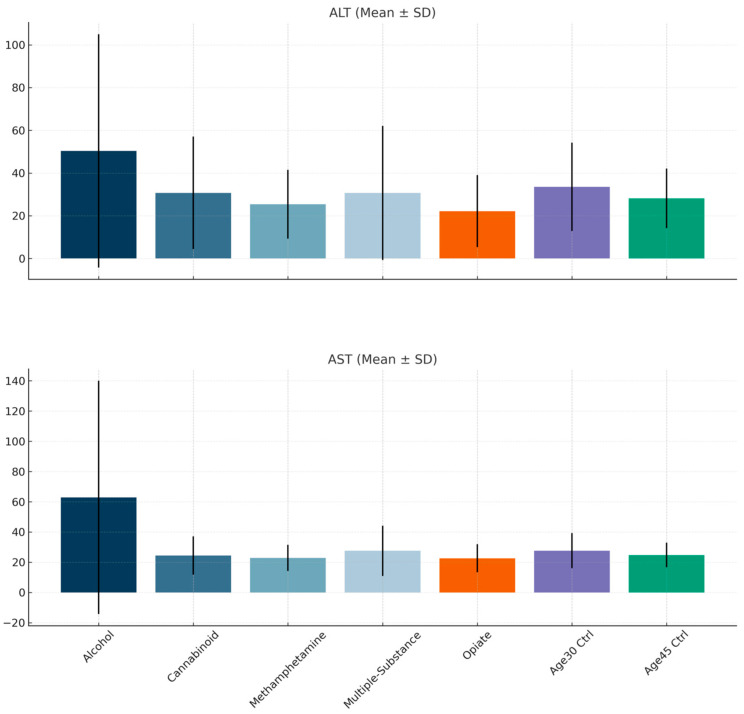
Comparison of groups in terms of liver enzymes.

Similarly, comparison of AST levels showed a significant group difference (H = 84.174, *p* < 0.001). Post hoc analyses indicated statistically significant differences between the opioid and 30-year-old control (*p* = 0.05), methamphetamine and 30-year-old control (*p* = 0.005), opioid and alcohol, methamphetamine and alcohol, cannabinoid and alcohol, multiple-substance and alcohol, 45-year-old control and alcohol (all *p* < 0.001), and 30-year-old control and alcohol (*p* = 0.001) groups (Figure 1).

When the groups were evaluated for anti-HCV seropositivity, a significant difference was detected (Fisher’s Exact Test, *p* < 0.001). Cross-tabulation analyses showed that anti-HCV positivity occurred exclusively in the opiate-use group (*n* = 9), whereas no positive cases were identified in any other groups. No significant differences were found among the groups in terms of HBsAg positivity (Fisher’s Exact Test, *p* = 0.671). There were no significant differences between the groups in terms of anti-HIV (Fisher’s Exact Test, *p* = 0.074).

### 3.3. Comparison of Blood Coagulation Parameters Between Substance Abusers and Control Groups

When the groups were compared in terms of serum calcium levels, a significant difference was observed (H = 18.756, *p* = 0.005). However, statistical significance was not retained in the post hoc pairwise comparisons (*p* > 0.05).

**Figure 2:**
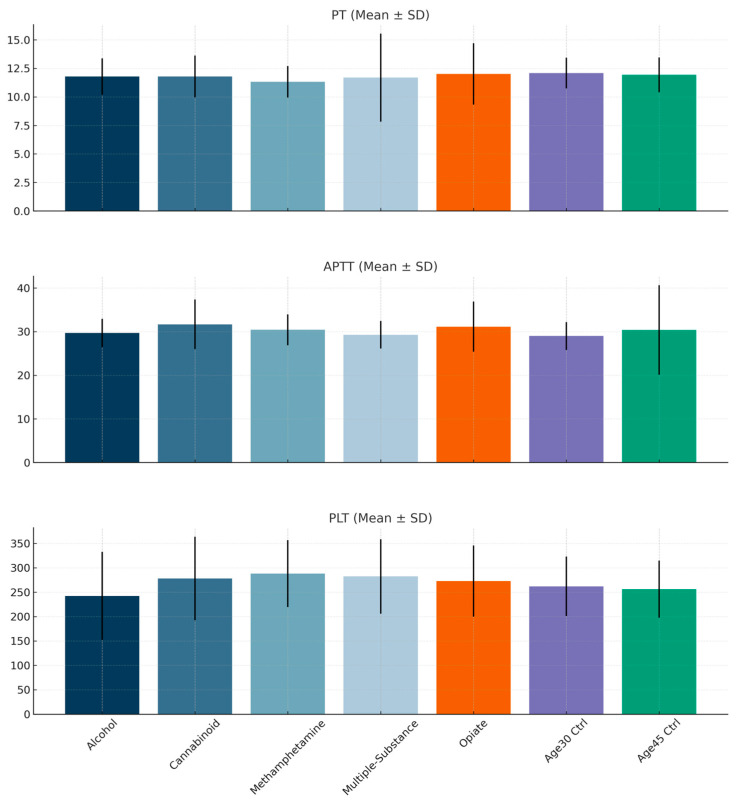
Comparison of groups in terms of coagulation factors.

Analysis of prothrombin time (PT) values revealed a significant difference among the groups (H = 27.357, *p* < 0.001). Post hoc tests indicated statistically significant differences between the multiple-substance and 30-year-old control groups, and between the methamphetamine and 30-year-old control groups (both *p* < 0.001) (Figure 2).

When the groups were compared in terms of activated partial thromboplastin time (aPTT) values, a significant difference was found (H = 27.158, *p* < 0.001). Post hoc analyses revealed statistically significant differences between the 30-year-old control and cannabinoid (*p* = 0.004), 45-year-old control and cannabinoid (*p* = 0.007), and multiple-substance and cannabinoid (*p* = 0.033) groups.

Comparison of platelet (PLT) values also showed a significant group difference (H = 26.771, *p* < 0.001). Post hoc analyses demonstrated significant differences between the alcohol and multiple-substance (*p* = 0.006) and alcohol and methamphetamine (*p* < 0.001) groups.

No statistically significant difference was found among the groups in terms of INR values (*p* = 0.164) (Table 2).

### 3.4. Association Between Smoking Status and Diagnostic Groups

A significant association was found between diagnostic groups and smoking status (χ^2^(6) = 230.59, *p* < 0.001). All individuals in the patient groups diagnosed with alcohol use disorder, multiple substance use disorder, cannabinoid use disorder, methamphetamine use disorder, or opioid use disorder were smokers. In the control groups, 46% of participants in the 45-year control group and 61% of those in the 30-year control group were smokers, corresponding to 535 smokers (88.9%) and 66 non-smokers (11.1%) in the total sample. Post hoc adjusted residual analysis revealed that the proportion of smokers was significantly higher across all patient groups compared to the control groups.

### 3.5. Comparison of Laboratory Parameters Between Smokers and Non-Smokers

No significant differences were observed between smokers (*n* = 535) and non-smokers (*n* = 66) in terms of ALT, AST, calcium, PT, aPTT, INR, or PLT levels (*p* > 0.05 for all comparisons). Although smokers exhibited slightly higher mean values for AST, INR, aPTT, and PLT, these differences did not reach statistical significance (Table 3).

## 4. Discussion

The main findings of our study are as follows: Compared to the control group of 30-year-olds, prothrombin time (PT) was lower in the Multiple-Substance and Methamphetamine groups. Additionally, activated partial thromboplastin time (APTT) values were higher in the Cannabinoid group. Among the substances studied, aspartate aminotransferase (AST) levels and alanine aminotransferase (ALT) levels were lower in both the Methamphetamine and Opiate groups. Furthermore, AST levels in the Alcohol group were elevated compared to the healthy control group aged 45. We also observed significant differences in AST, ALT, PT, APTT, and platelet (PLT) values based on the type of substance used by individuals.

Our findings indicate that PT values were lower in both the Multiple-Substance and Methamphetamine groups when compared to the 30-year-old control group. This shortening of PT may predispose individuals to serious health issues, such as thrombosis, myocardial infarction, stroke, or deep vein thrombosis [23]. Research has indicated that methamphetamine users face an increased risk of thrombosis, resulting in a higher likelihood of cardiovascular complications such as acute myocardial infarction [24,25]. A study by Sharma et al. also found that cocaine use, a stimulant like methamphetamine, contributes to a prothrombotic condition [26]. These findings align with our study, highlighting the need for caution regarding thrombotic conditions and associated cardiovascular complications in these patients. In the Multiple Substance group, the short prothrombin (PT) duration observed can be interpreted as consistent with the aforementioned literature, given the use of methamphetamine among these individuals. Furthermore, the presence of cannabinoid use in the Multiple Substance group adds complexity to the analysis. Therefore, further research examining the specific amounts of methamphetamine and cannabinoid use in these patients would be beneficial.

In the context of APTT values, the findings indicate that the APTT values in the cannabinoid group were significantly higher than those observed in the control group. A study conducted by Levendal and Frost demonstrated that the administration of a plant-based cannabinoid extract to Wistar rats resulted in an increased clotting time [27]. In addition, contemporary research has established a connection between cannabis use and conditions such as hemoptysis and alveolar hemorrhages, which appear to be consequences of its anticoagulant effects [28,29]. Conversely, it is important to note that there are discordant studies that suggest cannabinoid use may exert procoagulant effects, ultimately leading to thrombosis [30,31]. When analyzing the current data in conjunction with the existing literature, it is evident that cannabinoids may contribute to APTT prolongation associated with anticoagulant properties. Consequently, it is advisable to assess patients utilizing cannabinoids with respect to their bleeding risk. Given the contradictory evidence surrounding the relationship between cannabinoids and coagulation, further targeted and advanced investigations are warranted.

In addition, research examining the effects of opiate use on coagulation factors has revealed no significant association between opiate use and levels of prothrombin time PT, APTT, or INR, a finding that aligns with our results [32,33].

Regarding PLT (platelet) values, the alcohol group exhibited lower values compared to the multiple-substance group and the methamphetamine group, while no significant difference was observed when compared to the control group. Research indicates that alcohol consumption can lead to a decrease in platelet count and aggregation, but there are also studies suggesting that acute alcohol intake may increase platelet activation [34,35]. Although these findings seem contradictory in light of our data, it is evident that alcohol influences platelet function, and further research in this area would be advantageous.

In terms of AST values, the alcohol group had higher readings than the opiate group, methamphetamine group, cannabinoid group, multiple-substance group, and control groups. Moreover, AST values in the methamphetamine and opiate groups were lower compared to those in the 30-year-old control group. For ALT values, the alcohol group showed higher levels compared to the methamphetamine, multiple-substance, and opiate groups; however, there was no significant difference between the alcohol group and the control group. A study by Harris and colleagues indicated that liver function test values tend to increase after alcohol consumption [36]. Consistent with our findings, many studies have established that AST is more closely associated with alcohol consumption than ALT [37]. A high AST/ALT ratio is known to indicate advanced alcoholic liver disease [38]. Studies have demonstrated that methamphetamine use can lead to liver damage through mechanisms such as oxidative stress, inflammation, and disruption of the cell cycle [39,40]. An experimental study by Atici et al. also found that morphine addiction elevated liver function test values [41]. In contrast, a case–control study by Bazmi et al. found that liver dysfunction was less common in the group using opioids [42]. When integrating these findings from the literature with our results, it can be concluded that alcohol consumption, in particular, contributes to liver damage. The presence of conflicting data in the literature regarding the effects of methamphetamine and opiate use on the liver, together with our findings, can be explained by the fact that liver enzyme levels are influenced by factors such as the duration of substance use, nutritional status, and muscle mass [42]. Careful monitoring of liver function in these patients would also be beneficial.

A significant association was found between smoking status and diagnostic groups, as all individuals with substance use disorders were regular smokers, while approximately half of the control participants reported smoking. Despite this high prevalence, no significant differences were observed between smokers and non-smokers in liver enzyme or coagulation parameters (*p* > 0.05 for all). This finding aligns with prior research showing that smoking is highly prevalent among individuals with substance use and psychiatric disorders [43], yet its direct biochemical effects are generally modest.

Although nicotine exposure has been implicated in influencing hepatic metabolism and hemostatic regulation through mechanisms involving oxidative stress, endothelial activation, and changes in platelet aggregation [44,45,46], human evidence suggests these effects are generally mild and not clinically significant in otherwise healthy individuals. Recent data indicate that cigarette smokers may show minor elevations in certain liver or coagulation markers, but findings remain inconsistent and of limited physiological relevance [45]. Accordingly, the absence of significant differences in ALT, AST, PT, aPTT, INR, and PLT between smokers and non-smokers in our study supports the interpretation that nicotine-related systemic effects are modest. Therefore, the biochemical alterations observed among the diagnostic groups are more likely attributable to substance use rather than to smoking itself.

In our study, the average age in the Alcohol group was found to be higher compared to other groups using substances. No significant age differences were observed among groups using drugs. In a study by Stevens and colleagues on this subject, similar to our findings, it was shown that trying various substances other than alcohol is more common among young people, while alcohol is preferred over other substances among older people [47]. In our study, more appropriate comparisons were made by selecting a control group with similar characteristics to the Alcohol group and a control group with similar characteristics to the substance groups’ average age.

This study has several limitations. Its retrospective and non-randomized design limits the ability to establish causal inferences. The quantity and duration of substance use were not precisely quantified, and the degree of nicotine dependence or the number of cigarettes smoked per day was not assessed, which may have influenced some biochemical parameters. In addition, for illicit substances such as methamphetamine, cannabinoids, and opiates, reliable quantitative dose information is rarely available in routine clinical records, which is a common methodological limitation in substance use research [48,49,50]. Because detailed information on cumulative dose and exact time since last substance use was not available for all patients, early withdrawal-related physiological and biochemical alterations may have influenced the measured liver and coagulation parameters, even though all biochemical assessments were performed on the first day of admission to the detoxification unit, and this should be considered when interpreting our findings [51,52]. Additionally, the exact proportions of methamphetamine and cannabinoid use within the multiple-substance group remain uncertain, and laboratory parameters were evaluated only at admission without follow-up measurements. Finally, because this was a single-center, retrospective study, the generalizability of our findings may be limited. In collaboration with other relevant centers in the future, prospective multicenter studies are needed to increase the generalizability and to confirm our results.

However, the study also has notable strengths. It was conducted in a specialized clinic with well-organized patient records, involved a relatively large and well-characterized clinical sample, and included two age-matched control groups, allowing robust comparisons across diagnostic categories. Furthermore, it is one of the few studies to examine liver and coagulation parameters simultaneously across multiple substance-use groups while also accounting for smoking status. These features, together with the standardized biochemical assessments at baseline, enhance the reliability, interpretability, and clinical relevance of the findings.

Despite several limitations, our study provides a unique contribution to the literature by demonstrating substance-specific alterations in both liver and coagulation parameters within the same clinical population. Notably, the identification of significantly lower PT values in methamphetamine and multiple-substance users and prolonged aPTT in cannabinoid users highlights differential haemostatic risks associated with specific substances. Additionally, the finding that smoking status did not significantly affect biochemical outcomes strengthens the interpretation that the observed alterations were primarily substance-related rather than nicotine-induced.

In order to clarify these relationships and determine the specific mechanisms underlying substance-related liver and hemostatic changes, future studies with prospective, randomized controlled, and biomarker-based designs that also consider the amount and duration of substance use and other possible factors are necessary.

## 5. Conclusions

Liver and coagulation parameters differed according to the predominant substance used among individuals with Alcohol or Substance Use Disorders. Lower PT values in Methamphetamine and Multiple Substance may be associated with an increased thrombosis risk, whereas prolonged aPTT in Cannabinoid users may indicate a bleeding tendency. It would be beneficial to carefully monitor this patient population for thrombosis or bleeding, depending on their drug of choice. Elevated AST and ALT levels in Alcohol users highlight the need for regular liver monitoring.

## Figures and Tables

**Table 1:** Comparison of demographic characteristics, liver functions, and HBV, HCV, and HIV between substance abusers and control groups.

		Group							*p*-Value
Parameters		Alcohol (*n* = 110)	Cannabinoid (*n* = 71)	Methamphetamine (*n* = 110)	Multiple-Substance (*n* = 110)	Opiate (*n* = 50)	Age 30 Control (*n* = 100)	Age 45 Alcohol Control (*n* = 50)	
**Age, Mean ± SD**		45.67 ± 11.44	30.73 ± 7.51	28.84 ± 6.05	30.93 ± 7.37	33.26 ± 8.01	29.90 ± 5.47	45.38 ± 2.64	**<0.001**
**Gender**	**Female**	5 (4.5%)	2 (2.8%)	9 (8.2%)	6 (5.5%)	7 (14.0%)	10 (10.0%)	5 (10.0%)	0.178
	**Male**	105 (95.5%)	69 (97.2%)	101 (91.8%)	104 (94.5%)	43 (86.0%)	90 (90.0%)	45 (90.0%)	
**ALT, Mean ± SD**		50.37 ± 54.62	30.70 ± 26.33	25.35 ± 16.09	30.68 ± 31.35	22.16 ± 16.84	33.51 ± 20.69	28.16 ± 13.97	**<0.001**
**AST, Mean ± SD**		62.85 ± 77.19	24.41 ± 12.69	22.84 ± 8.59	27.58 ± 16.63	22.64 ± 9.27	27.68 ± 11.59	24.80 ± 8.07	**<0.001**
**Anti** **HCV**	**S_+_**	0	0	0	0	9	0	0	**<0.001**
	**S_−_**	110	71	110	110	41	100	50	
**HBs AG**	**S_+_**	3	2	2	3	0	4	3	0.671
	**S_−_**	107	69	108	107	50	96	47	
**Anti** **HIV**	**S_+_**	0	0	3	0	0	0	0	0.074
	**S_−_**	110	71	107	110	50	100	50	

Continuous variables were analyzed using one-way ANOVA for normally distributed data and the Kruskal–Wallis test for non-normally distributed data. Categorical variables were compared using the chi-square test. Bold values indicate statistically significant results (*p* < 0.05).

**Table 2:** Comparison of blood coagulation parameters between substance abusers and control groups.

	Group							*p*-Value
	Alcohol (*n* = 110)	Cannabinoid (*n* = 71)	Methamphetamine (*n* = 110)	Multiple-Substance (*n* = 110)	Opiate (*n* = 50)	Age 30 Control (*n* = 100)	Age 45 Alcohol Control (*n* = 50)	
**Ca, Mean ± SD**	9.46 ± 0.51	9.66 ± 0.41	9.58 ± 0.39	9.60 ± 0.39	9.45 ± 0.40	9.62 ± 0.40	9.46 ± 0.32	**0.005**
**PT, Mean ± SD**	11.78 ± 1.60	11.79 ± 1.83	11.32 ± 1.38	11.69 ± 3.86	12.01 ± 2.69	12.09 ± 1.34	11.93 ± 1.52	**<0.001**
**APTT, Mean ± SD**	29.68 ± 3.23	31.67 ± 5.68	30.41 ± 3.55	29.27 ± 3.14	31.13 ± 5.74	29.01 ± 3.20	30.39 ±10.27	**<0.001**
**PLT, Mean ± SD**	242.37 ± 90.14	278.15 ± 85.76	288.05 ± 68.50	282.19 ± 76.07	272.72 ± 73.15	262.08 ± 61.18	256.00 ± 58.59	**<0.001**
**INR, Mean ± SD**	1.17 ± 0.57	1.11 ± 0.14	1.07 ± 1.10	1.14 ± 0.38	1.14 ± 0.27	1.09 ± 0.12	1.09 ± 0.15	0.164

Data are presented as mean ± standard deviation. Group differences were assessed using the Kruskal–Wallis test. Statistically significant results are shown in bold (*p* < 0.05).

**Table 3:** Comparison of laboratory parameters between smokers and non-smokers.

Parameter	Non-Smokers (Mean ± SD) *n* = 66	Smokers (Mean ± SD) *n* = 535	U	*p*
ALT (U/L)	32.15 ± 21.28	32.95 ± 33.33	15,864.5	0.178
AST (U/L)	26.62 ± 11.74	32.85 ± 39.60	17,534.5	0.928
Calcium (mg/dL)	9.60 ± 0.43	9.56 ± 0.42	16,995.0	0.619
PT (s)	11.92 ± 1.52	11.75 ± 2.33	15,598.0	0.122
aPTT (s)	29.44 ± 6.31	30.12 ± 4.64	15,275.5	0.074
INR	1.08 ± 0.14	1.12 ± 0.34	15,616.0	0.125
PLT (×10^3^/µL)	261.50 ± 56.22	270.13 ± 78.51	16,624.5	0.439

Data are presented as mean ± standard deviation. The Mann–Whitney U test was used for comparisons between smokers and non-smokers. No statistically significant differences were observed between groups (*p* > 0.05).

## Data Availability

The data presented in this study are available on request from the corresponding author due to ethical approval requirements.

## Abbreviations

The following abbreviations are used in this manuscript:

AUD	Alcohol Use Disorder
SUD	Substance Use Disorder
PT	Prothrombin Time
aPTT	Activated Partial Thromboplastin Time
PLT	Platelet
